# Full, free, and informed: defining and operationalising informed choice for menstrual supplies

**DOI:** 10.1080/26410397.2025.2538357

**Published:** 2025-07-25

**Authors:** Lucy C. Wilson, Tanya Dargan Mahajan

**Affiliations:** a Independent Consultant, Rising Outcomes, Hillsborough, NC, USA. *Correspondence*: lucy.wilson@gmail.com; b Co-founder, Menstrual Health Action for Impact (MHAi), New Delhi, India

**Keywords:** menstrual health, choice, rights-based programming

## Abstract

Informed choice is a cornerstone of bodily autonomy and dignity, yet remains underdeveloped in menstrual health programmes and markets, particularly in low- and middle-income countries. Currently, menstruation-related stigma and gaps in access, availability, and education constrain individuals’ ability to choose menstrual supplies that best suit their needs. This article proposes a gender-transformative definition of informed choice for menstrual supplies, adapted from the “full, free, and informed” framework widely used in family planning. Informed choice for menstrual supplies means having access to the broadest possible range of supplies, the freedom to choose without stigma or coercion, and access to accurate, unbiased information. The range of menstrual supplies should include single-use and reusable products, contraception, pain relief, and supportive items. Pathways for operationalising this framework in both commercial and free distribution settings are explored, with recommendations for expanding choice, addressing systemic barriers, and improving menstrual literacy. Ultimately, enabling full, free, and informed choice in menstrual health can improve individual health outcomes, reduce stigma, enhance equity, and foster well-functioning markets responsive to the needs of all who menstruate.

## Résumé

Le choix éclairé est un élément fondamental de l’autonomie et de la dignité corporelles. Il reste pourtant peu développé dans les programmes et les marchés de santé menstruelle, en particulier dans les pays à revenu faible ou intermédiaire. Actuellement, la stigmatisation liée aux menstruations et les lacunes en matière d’accès, de disponibilité et d’éducation limitent la capacité des personnes à choisir les produits menstruels les mieux adaptés à leurs besoins. Cet article propose une définition sexotransformatrice du choix éclairé pour les produits menstruels, adaptée du cadre de choix « complet, libre et éclairé » largement utilisé dans la planification familiale. Un choix éclairé de produits menstruels suppose d’avoir accès à l’éventail le plus large possible de fournitures, d’être libre de choisir sans stigmatisation ni coercition et de disposer d’informations précises et impartiales. La gamme de produits menstruels devrait inclure des produits à usage unique ou réutilisables, des contraceptifs, des analgésiques et des articles de soutien. Les pistes pour mettre en œuvre ce cadre dans les contextes de distribution commerciale et gratuite sont étudiées, avec des recommandations pour élargir le choix, lever les obstacles systémiques et améliorer les connaissances élémentaires relatives aux règles. En fin de compte, permettre un choix complet, libre et éclairé en matière de santé menstruelle peut améliorer l’état de santé individuel, réduire la stigmatisation, renforcer l’équité et favoriser des marchés performants et réactifs aux besoins de toutes les personnes qui ont des menstruations.

## Resumen

La elección informada es un pilar de la autonomía corporal y la dignidad; sin embargo, continúa subdesarrollada en los programas y mercados de salud menstrual, en particular en países de bajos y medianos ingresos. Actualmente, el estigma relacionado con la menstruación y las lagunas en acceso, disponibilidad y educación limitan la capacidad de las personas para elegir suministros menstruales que sean los más indicados para sus necesidades. Este artículo propone una definición transformadora del género de la elección informada de suministros menstruales, adaptada del marco “completo, libre e informado” utilizado de manera generalizada en planificación familiar. La elección informada de suministros menstruales significa tener acceso a la gama más amplia posible de suministros, la libertad para elegir sin estigma o coerción, y el acceso a información veraz y sin prejuicios. La gama de suministros menstruales debe incluir productos de uso único y reutilizables, métodos anticonceptivos, medicamentos para aliviar el dolor y artículos de apoyo. Se exploran las vías para operacionalizar este marco tanto en ámbitos comerciales como en ámbitos de distribución gratuita, y se ofrecen recomendaciones para ampliar las opciones, abordar barreras sistémicas y mejorar la alfabetización menstrual. A la larga, al facilitar la elección completa, libre e informada en salud menstrual, se mejoran los resultados de salud individual, se reduce el estigma, se realza la equidad y se fomentan mercados en buen estado de funcionamiento que responden a las necesidades de todas las personas que menstrúan.

## Background

An individual's ability to choose what is right for their body is a fundamental human right. It is especially important in the context of sexual and reproductive health, including menstrual health (MH). Allowing individuals to choose the supplies with which they want to manage their menstruation supports their autonomy and dignity. Within the MH field, there are growing calls for informed choice.^1–5^ There is, however, no common understanding of what informed choice for menstrual supplies^1^ means, and little guidance on how it can be operationalised.^1^

As such, MH programme planners, especially those working on product distribution, still ask “What is the best product to provide?”, rather than, “How can we offer a range of options to best meet people’s needs?” And MH markets in low- and middle-income countries (LMICs) often offer a limited array of options, as a lack of investment on the supply side, lack of awareness on the demand side, and other barriers limit market entry.^4,6^

Observation of menstrual supply markets, policies, and programmes over the last decade led the authors to propose this definition and contextualisation for informed choice for menstrual supplies. In particular, we have been struck by the differences in how the concept of informed choice is realised (or not) across contexts, including between high-income countries and LMICs and among LMICs with varying levels of market openness. In our observation of programmes, the differences in how stakeholders in the global MH and family planning fields have approached supply provision and informed choice have also motivated our development of this definition. LW is an independent consultant based in and from the Global North (USA) who has worked on global family planning programmes for nearly 20 years and on menstrual health for 10 years. TM is based in the Global South (in India) with 15 years of experience working at the intersection of policy, research, and markets for public health and uses evidence-based strategies to support development of menstrual health markets in low- and middle-income countries.

This article proposes a gender-transformative^2^ definition of informed choice for menstrual supplies. It begins with background on the status of informed choice in menstrual supply markets and programmes and then outlines the types of menstrual supplies. It covers the importance of expanding both the array of options and an individual’s ability to choose among those options. Finally, this article explores pathways for operationalising choice using the full, free, and informed choice approach in commercial markets and distribution programs. In summary, this article argues that informed choice for menstrual supplies, including improved education and a broader range of options, will increase use of safe and good quality supplies and contribute to the health, dignity and well-being of girls, women and others who menstruate.

## Status of choice

Estimates indicate 35% and 72% of those who menstruate in low- and lower-middle-income countries, respectively, use purpose-made (commercially available) products to manage their bleeding.^7^ Most LMIC markets offer few if any options beyond single-use (i.e. disposable) pads. Some users receive free or subsidised products through programmes run by governments, non-profits, or non-governmental organisations (NGOs). These programmes also tend to offer only one product option, with that option varying by programme.

Only those in the highest wealth quintiles are able to access and afford a continued supply of single-use purpose-made products, leading to inequity in access.^8^ In addition, girls, women, and others who menstruate may have limited ability to freely choose their menstrual supplies because another family member controls the finances or is controlling or influencing menstrual supply decisions for the household.^4,9,10^ Access to sanitation infrastructure can also affect a person’s ability to use their preferred menstrual supply.^9,10^ Marginalised groups, including those with disabilities and displaced persons, may face greater barriers to access and choice.^11,12^

Low awareness of the full range of menstrual supplies also limits choice.^2,13^ For example, in a study conducted with 407 menstruating people in rural and peri-urban India, only 41% of menstruators were aware of reusable pads, 17% of reusable menstrual underwear, and 4% of menstrual cups.^14^

Stigma associated with menstruation, especially stigma associated with specific menstrual supplies, further limits choice. Repurposed and new cloth can be an appropriate and hygienic option, however, cloth can be stigmatised as unhygienic and only for those without resources. Similarly, reusable pads and menstrual underwear may be dismissed as inferior to single-use options.^15^ Vaginal insertion products have been stigmatised due to misconceptions about virginity.^16^ Contraception, an option to help manage menstrual bleeding and pain, is subject to misconceptions about safety and suitability, especially for younger menstruators. Finally, criticism of single-use pads as harmful for the environment may shame those who choose to use them.^17^

Governments, donors, and others in LMICs who manage distribution programmes and commercial market actors adapt to these stigmas by only offering products that are considered socially acceptable. When programmes and markets oblige stigmas, they accommodate gendered social norms that limit options for girls, women, and others who menstruate. When users of specific products are shamed or vilified, these gendered stigmas and norms are exploited. On the other hand, challenging these stigmas by providing information and offering the fullest possible range of options to manage menstruation can be gender-transformative.^3^

## Broadening the definition of menstrual supplies

In the past, defining informed choice for menstrual supplies has been complicated by an inconsistent understanding of the supply options. Here, we propose a definition for menstrual supplies for managing menstrual bleeding and other symptoms:

- Products that collect or absorb menstrual fluids including single-use and reusable purpose-made products such as pads, liners, tampons, menstrual underwear, cups or discs, and repurposed cloth.
- Hormonal contraceptives that increase the predictability of bleeding, reduce duration or volume, or help to manage pain and other menstrual symptoms.
- Over-the-counter analgesics for pain and other symptoms.
- Supportive supplies needed to appropriately use, wash, dry, and store the product(s) used to manage bleeding, e.g. underwear and soap.

An even broader range of products, supplies, and services, including for diagnosis, counselling, self-care, and treatment may be needed to fully address the needs of menstruating people. However, they fall beyond the scope of our paper.

## Importance of choice

The primary reason that informed choice for menstrual supplies is important is that having a wider range of options available increases the likelihood that each person will find an option that meets their needs and preferences. Research and data, while limited, consistently show that there is no single best menstrual management option for all people. For instance, a 2018 study in Kenya offered adolescent girls a choice between 30 single-use pads, two reusable pads, and one menstrual cup, and 51% choose reusable pads, 46% choose single-use pads, and 3% choose the menstrual cup.^3^ Other research has also shown a diversity of preferences. People may use multiple bleeding management options, and preferred options vary over time and circumstance.^4,5,9,10,18^ They may use cloth at home and single-use pads at work, a cup and a pad at the same time, or single-use pads during the dry season and reusable pads when there is ample water.

There are also potential indirect benefits from full, free, and informed choice, including increased knowledge and body literacy, which may lead to healthier behaviours and fewer urogenital tract infections (UTIs). To inform uptake of novel options, programmes and commercial markets that offer a choice of menstrual supplies must provide more information on menstrual health than those that offer only a single option. For instance, washable pad use requires messages that menstrual blood is not impure and menstrual cup use requires familiarity with female reproductive anatomy. This increased information may lead to improved healthy and health-seeking behaviours.^19,20^

When users are not able to adequately change their menstrual product, using it for longer than its recommended duration, they are more likely to acquire a UTI.^21,22^ Access to a wider choice of supplies and appropriate information may enable people to find an option(s) they can afford to change (wash and dry or replace) more frequently, with the potential to decrease the incidence of UTIs.

Other potential indirect benefits of informed choice exist at a macro level. Expanding the range of options has the potential to increase greater overall use and more diversified use of safe, effective, high-quality menstrual supplies, as was seen in the family planning field. A review of data from more than 100 countries across almost thirty years showed that as the number of contraceptive options in a country increased, there was a correlated increase in overall contraceptive use.^23^ Some existing contraceptive users switched to the newly offered method, but non-users also began contracepting with the introduction of the new method. Several MH-focused studies have replicated this finding on a smaller scale, with some participants switching to newly offered option(s) and some sticking to their existing option(s).^2,24^

As more choice is offered to girls, women and others who menstruate, there will likely also be increased demand for higher quality products and increased competition amongst suppliers, resulting in overall improved quality of menstrual supplies. Increased competition can also lead to lower prices and thus improved affordability.^25^ Diversification may make supply chains more resilient by decreasing pressures on specific products.^26^ Importantly, it may also lessen the environmental burden of single-use products.

## Defining informed choice for menstrual supplies

Within the family planning field, the term “full, free, and informed choice” is often used.^27–29^ This three-prong framework to choice, which builds from the field of medical ethics, was defined in 2012 to mean access to the full (or widest possible) range of contraceptive methods; through a choice made voluntarily, free from barriers or coercion; and with information that is complete, accurate, and unbiased about all methods.^30^

Our definition^4^ of informed choice for menstrual supplies extrapolates from this full, free, and informed framework. In the MH context, informed choice means:

- Access to the fullest possible range of menstrual supply options, including contraception, pain management, and supportive supplies
- Access to these supplies in a manner that is free from stigma, pressure, and barriers; and
- Access to information that is accurate, unbiased, and sufficient for decision-making and use by the individual.

**Figure 1.**
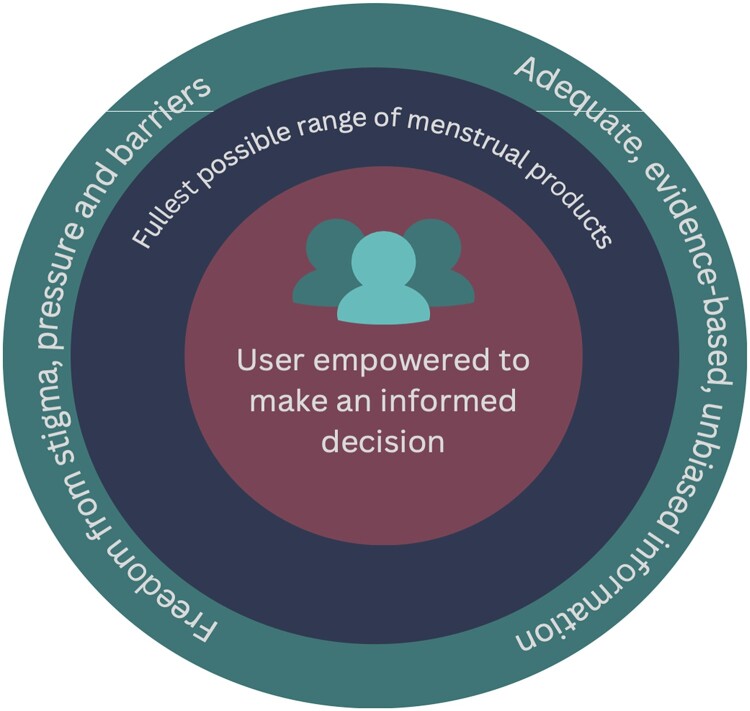
Framework for full, free, and informed menstrual choice.

This definition – and the full, free, and informed choice for menstrual supplies framework (Figure 1) – is applicable both to commercial markets (and market-shaping interventions) and programmes that distribute products and supplies and share information.

Ensuring that everyone has full, free, and informed choice for menstrual supplies should improve their individual ability to choose and use the supplies that work best for them. However, there are some barriers to choice that may not be adequately addressed. For instance, limited individual agency, access to financial resources, and access to sanitation infrastructure may all still limit choice. In addition, while more information and fewer barriers can help reduce stigma, the stigmas associated with menstruation are deep and may continue to create barriers.

## Operationalising informed choice for menstrual supplies in commercial markets

Full, free, and informed choice for menstrual supplies can support the growth of and serve as a benchmark for a healthy and well-functioning menstrual health market. This can be operationalised as:

- **Full:** While a single retail location may not be able to carry the full range of supplies, the fullest possible range should be available within the local market. While innovations may take time to reach local markets, especially in LMICs, policies and programmes should facilitate their market entry and encourage a broad and competitive market. For instance, tax policies and quality standards that apply to only one product type should be made applicable to all menstrual bleeding products or the broader range of supplies.
- **Free:** Sales agents, counsellors, educators, and other market actors should operate in a way that reduces pressure and stigma, especially product-related stigma. Individuals should be able to choose supplies based on their own needs and preferences. In addition, barriers to choice at the systems level need to be removed. For instance, reforms should be undertaken if menstrual products are taxed unevenly or if certain product types are excluded from supportive policies.
- **Informed:** Market actors, including commercial actors like manufacturers, distributors, retailers, marketeers, and non-commercial actors like governments, NGOs, social enterprises, and other advocates, can play a role in enabling informed choice by providing menstrual education and category-wide product and supply promotion. The information must be adequate for decision-making, including unbiased information on the potential advantages, disadvantages, and proper use and maintenance. It should also aim to dismantle stigmas and biases rooted in social norms. Brand owners and suppliers must promote products honestly, without stigmatising competitor products or supplies. Finally, people have the right to accurate information about the full range of options, even if they are not available locally. With information, individuals can seek out supplies elsewhere or advocate for their market entry.

## Operationalising menstrual choice in distribution programmes

To ensure equity for those not well-served by the market, governments, non-profits, and social enterprises may offer supplies through free or subsidised distribution programmes. Menstrual product distribution programmes can adopt full, free, and informed choice by supplementing distribution with additional supplies, information, and referrals.

- **Full:** The fullest possible range of options should be offered. Programmes should endeavour to provide at least two different types of bleeding management products along with supportive supplies. For options not offered, the programme should identify sources so that referrals can be made. Referrals should include collaborations for subsidised access and linkages to pharmacies and health care facilities for analgesics and contraception.
- **Free:** Participants should be free to choose their preferred option(s) without pressure, shame, or product-related stigma.
- **Informed:** Many distribution programmes already include some menstrual education and information. To enable choice, this should include accurate unbiased information on the full range of supply options. The information must be adequate for decision-making, including information on the potential advantages, disadvantages, and how to maintain, use, and dispose of products and supplies, including non-purpose-made options and those not offered through the programme.

While we have historically seen programmes choose to focus on a single product, some are already offering unbiased information and multiple product options.^5^ Programmes that integrate MH across other health areas, including sexual and reproductive health, may be best placed to offer menstrual choice as the existing approach towards informed choice and the existing structures may offer an opportunity to diversify the available supplies and services offered.^31^

## Conclusion

Despite growing access to menstrual products in LMICs, in most contexts there remain only one or two options to manage menstrual bleeding. A broader range of options has the potential for greater satisfaction, dignity, and well-being for girls, women and others who menstruate. **Full, free, and informed menstrual choice** is needed to ensure that these ideals are met. This three-pronged framework for choice means that users – rather than programme planners, funders, and market actors – choose the supplies they will use to manage their menstruation.

We are not aware of any context, in either a commercial market or a distribution programme, where full, free, and informed choice currently exists, but we hope that by providing this definition, more targeted efforts to achieve such conditions can be undertaken. In addition, research is needed to test and validate approaches to increase informed menstrual choice and to understand the implications of greater choice on other aspects of their life and health. In the meantime, we need advocacy to ensure full, free, and informed menstrual choice in programmes and markets. This is essential for providing equitable access for all people who menstruate across LMICs.

## Footnotes

1 We use “products” to include purpose-made products and repurposed materials used to manage menstrual bleeding. We use “supplies” to encompass the wider range of options available to manage menstruation, including bleeding management products, contraceptives, pain management supplies, and supportive supplies. A more detailed definition is provided on the subsequent page.

2 See the USAID Gender Integration Continuum. https://www.igwg.org/training/programmatic-guidance/.

3 See the USAID Gender Integration Continuum. https://www.igwg.org/training/programmatic-guidance/.

4 We adapted this definition based on our experiences, presented it for discussion at the 2023 Reproductive Health Supplies Coalition General Membership Meeting in a session of the Menstrual Health Supplies Workstream, and made further changes based on discussions at the meeting.

5 For example, the “Know your options” toolkit from the Reproductive Health Supplies Coalition: https://www.rhsupplies.org/activities-resources/groups/newunderused-rh-technologies-caucus/menstrual-health-supplies-workstream/menstrual-health-supplies-options/.

## Provenance statement

This article was not commissioned and went through external peer review.
